# Breast reconstruction during the COVID-19 pandemic in resource-limited settings

**DOI:** 10.3205/iprs000160

**Published:** 2021-09-08

**Authors:** Sammy Al-Benna, Andreas Gohritz

**Affiliations:** 1 Division of Plastic and Reconstructive Surgery, Faculty of Medicine and Health Sciences, Stellenbosch University and Tygerberg Academic Hospital, Cape Town, South Africa; 2 Department of Plastic, Reconstructive, Aesthetic and Hand Surgery, University Hospital Basel, Switzerland

**Keywords:** reconstructive surgical procedures, mammaplasty, breast implantation, surgical flaps, severe acute respiratory syndrome coronavirus 2, COVID-19, pandemics

## Abstract

The coronavirus disease 2019 (COVID-19) is a novel, rapidly changing pandemic. It has affected specialized medical services in unprecedented ways. Surgical decision making, always the most important aspect of care has taken on an added layer of complexity in the face of the COVID-19 pandemic. Therefore, recommendations for breast reconstruction during COVID-19 remain challenging and unclear. This article reviews the impact of the COVID-19 pandemic and suggests potential approaches that could be considered in the absence of validated strategies in breast reconstruction.

## Zusammenfassung

COVID-19 (Coronavirus Disease 2019) ist eine neuartige, sich schnell verändernde Pandemie. Sie hat spezialisierte medizinische Dienstleistungen in nie dagewesener Weise beeinflusst. Die chirurgische Entscheidungsfindung, immer der wichtigste Aspekt der Versorgung, hat angesichts der COVID-19-Pandemie eine zusätzliche Ebene der Komplexität angenommen. Daher bleiben die Empfehlungen für die Brustrekonstruktion während COVID-19 herausfordernd und unklar. Dieser Artikel gibt einen Überblick über die Auswirkungen der COVID-19-Pandemie und schlägt mögliche Ansätze vor, die in Ermangelung validierter Strategien bei der Brustrekonstruktion in Betracht gezogen werden könnten.

## Introduction

Severe acute respiratory syndrome coronavirus 2 (SARS-CoV-2) is an enveloped, positive-sense, single-stranded RNA β-coronavirus [1]. SARS-CoV-2, which causes the disease known as coronavirus disease 2019 (COVID-19), was first reported in late 2019 in Wuhan, China and has rapidly developed into a global pandemic and public health emergency [1], [2], [3]. As of 1^st^ September 2020, a total of 25,671,607 accumulated cases and 855,649 deaths have been reported worldwide, and South Africa is the current epicentre of the COVID-19 pandemic in sub-Saharan Africa [4], [5]. This pandemic places a strain on healthcare systems and providers, as well as forcing difficult choices about whether care can and should be delayed or reprioritized [5], [6]. The need to dedicate major economic, infrastructural, and medical resources to the assistance of critically ill COVID-19 patients is causing a redistribution of the activities of several medical disciplines not primarily involved in the management of COVID-19 patients [5], [6]. Given the varying natures of international healthcare systems, conditions differ significantly by locality; critical decisions concerning the deployment of resources and the management of elective surgical procedures should be made based on institutional policies and recommendations from local, state and federal authorities, considering the availability of finite and essential resources [2], [3], [5], [6]. Health care professionals have a responsibility to maximise the use of these resources to provide the best possible care for all patients. Patients must be informed of local and national service constraints and be given the opportunity to understand their planned treatment within allowable resources [2], [3], [5], [6], [7]. High level ACE2 gene expression has been demonstrated in skin, fat and breast tissue [8], [9], [10]. In addition, changes in wound healing pathophysiology and free flap failures have been associated with COVID-19 [11], [12]. This article focuses on the effects of the COVID-19 pandemic on breast reconstruction and suggests potential approaches that could be considered in the absence of validated strategies.

## Breast reconstruction prior to COVID-19

Breast cancer is the most common malignancy in women worldwide, with over 2 million new cases diagnosed per year, contributing over 25% to new cancer cases diagnosed (excluding non-melanoma skin cancer) [13]. There has been an increase in the incidence with a rate of up to 3.5% over the preceding decade [13]. Surgery remains the foundation of treatment for breast cancer worldwide, and current current trends in surgical care including breast conservation surgery, mastectomy and reconstruction [14], [15], [16], [17], [18], [19]. There has been a paradigm shift in the management of breast cancer and the advent of oncoplastic breast surgery has revolutionised the concept of breast preservation, utilising limited tissue resection as opposed to a mastectomy [20], [21], [22], [23], [24], [25], [26], [27]. Although breast conservation therapy can be an attractive alternative, it is dependent upon a number of clinical factors including tumour size and location, and importantly approximately 40% of women with primary breast cancer still undergo mastectomy [14], [27], [28], [29], [30], [31], [32], [33].

A diagnosis of breast cancer and subsequent treatments are stressful events in a patient’s life, associated with an increased risk of anxiety, depression and psychosocial implications [34], [35], [36]. The decision to proceed with a mastectomy can lead to a feeling of loss of feminity, issues around sexuality and self-esteem as well as practical problems associated around clothing and the wearing of a prosthesis [34], [35]. Accordingly, a growing mumber of women now seek immediate reconstruction after mastectomy [25], [26]. This may be seen as an integral addition to their breast cancer treatment, resulting in improvements to their appearance and general well-being. The beneficial effects of breast reconstruction have been widely reported, and the vast majority of women is satisfied with the overall outcome, irrespective of the type of procedure undertaken [35], [36].

The timing of breast reconstruction following a mastectomy is important as there are a number of pertinent issues that need to be taken into consideration when opting for an immediate versus delayed reconstruction [34], [35]. These include the patient's body form, general health, breast cancer management as well as patient's preference [14].

At most breast centres, the majority of reconstructions is performed immediately, in a joint procedure between the breast surgeon, who performs the mastectomy, and the plastic surgeon [15], [19], [31], [34], [35], [36], [37]. Pre-operatively, this allows for mastectomy skin sparing incisions to be discussed between the two teams which can then be incorporated in the final decision on the optimal reconstruction to best suit the patient. Immediate breast reconstruction confers many advantages, mostly associated with an improved aesthetic appearance. It can also reduce numerous incisional scars and the need for multiple operations as well as offering significant psychological benefit with the restoration of the breast.

A major concern that can arise from an immediate reconstruction is a potential delay in patients receiving adjuvant therapy, i.e. radiotherapy and/or chemotherapy whilst recovering from surgery. Multi-disciplinary teams involving the oncologists as well as the surgeons work together to prevent this occurring. However, if there are certain patient co-morbidities that could prolong the post-operative recovery period, then a delayed reconstruction may be a more viable option. usually a year after completion of adjuvant therapy. Unfortunately, radiotherapy or scar tissue due to the mastectomy may affect wound healing as well as the integrity of vessels critical for the survival of autologous reconstruction and patients will be made aware of this.

Delayed reconstruction is also an option for many patients who are overwhelmed with their recent diagnosis and respective surgical treatment [34], [35], [36]. lt gives them time to make a fully informed decision before choosing the type of reconstruction that would benefit them [34], [35], [36].

A third option of immediate-delayed reconstruction may provide an alternative solution for those patients awaiting histological confirmation and possible post-mastectomy radiotherapy [36]. In this situation, a saline tissue expander implant is placed at the time of mastectomy protecting the breast skin envelope and remains until the patient completes the radiation cycle [30], [32], [36]. They can then undergo the standard delayed reconstruction. If radiotherapy is not required, the implant can be exchanged for a reconstructive procedure at an earlier date [32].

## Autologous breast reconstruction

The aim of reconstruction is to recreate a breast “footprint” that is similar in shape, form and consistency to the natural/contralateral breast [16], [30], [35], [36]. Hence, breast reconstruction employs either autologous tissue or a non-autologous procedure to achieve a pleasing aesthetic result [7], [36].

Immediate breast reconstruction with autologous tissue is the preferred method, favoured by many patients and involves the microsurgical transfer of tissue from one area of the body to recreate the new breast. The type of flap is individualised to the patient’s needs as well as optimising the use of available tissue [14], [16]. At our institution, the most common flaps carried out are the deep inferior epigastric artery perforator (DIEAP) flap followed by the pedicled latissimus dorsi flap (LD) [14], [16], [38]. The gold standard for autologous reconstruction in the post-mastectomy patient remains the DIEAP flap, although many women may not be candidates for abdominally based free tissue transfer. In this scenario, there are several other donor site options on the thigh (transverse and diagonal upper gracilis flaps, profunda artery perforator flap, lateral thigh flap) and trunk (lumbar artery perforator flap, superior and inferior gluteal artery perforator flaps) [38], [39]. 

The DIEP flap, which is based on the deep inferior epigastric artery and vein, necessitates the presence of excess lower abdominal tissue. The procedure involves identification and meticulous dissection of the vessels as they course through the rectus abdominis muscle to the proximal pedicle of the external iliac artery and vein. The vessels are carefully isolated and transected together with excision of excess skin and subcutaneous tissue (referred to as a skin paddle) from the lower abdomen. The flap of tissue is then sculptured on the table to create a breast shape relative to the contralateral side before insetting into the new reconstructed breast. The flap is microsurgically anastomosed to the internal mammary or thoracodorsal vessels to re-establish blood perfusion.

Prior to the advent of the DIEAP free flap, the TRAM (transverse rectus abdominis myocutaneous) flap was the gold standard for breast reconstruction and involved a similar technique of harvesting the skin paddle but in addition to muscle from the lower abdomen. It can be associated with significant donor site morbidities including a reduced abdominal strength and development of an incisional hernia.

If the patient lacks adequate abdominal soft tissue, a transverse myocutaneous gracilis flap (TUG) can be reconstructed [40]. This recruits skin, subcutaneous tissue and muscle from the upper thigh which is based on the cutaneous gracilis perforators from the medial femoral circumflex system [40]. It is particularly advantageous as it provides minimal donor site morbidity and a scar that is acceptable to the patient [41].

The pedicled latissimus dorsi flap is a suitable alternative for those patients who are unwilling to accept abdominal weakness or who lack excess tissue [42]. It employs a small ellipse of skin and muscle from the back based on its own blood supply, and is advanced into the new breast via a dissected tunnel within the axilla. The use of part of the latissimus dorsi does not cause any adverse restrictions on shoulder function due to the synergistic effect of the rotator cuff muscles which can compensate for normal activities [43]. This flap was introduced at a time when radical mastectomy involved resection of the pectoralis major muscle with remnant thin skin flaps. The LD flap not only provides optimal soft tissue and muscle replacement to enhance the new breast shape but permits adequate coverage in the use of a breast implant [44].

Autologous reconstruction offers many benefits: It replaces like for like tissue, eliminates the need for implants in the majority of cases and creates a natural looking breast footprint [45]. Furthermore, the reconstructed tissue can alter in size, keeping with weight gain or loss and never requires replacement as seen with breast implants. Finally, many patients welcome the essential abdominoplasty that is required to harvest abdominal tissue for the DIEAP/TRAM flap.

Breast reconstruction is a complex procedure that is associated with complications which include bleeding, flap failure due to a compromised blood supply, fat necrosis of the new breast tissue and donor site morbidity, such as the development of a seroma or abscess collection. Unfortunately, this may necessitate the patient returning to the operating theatre to salvage the flap. It this is not possible, the dead tissue is removed and further options for reconstruction are discussed.

One of the major concerns highlighted is a potential risk for loco-regional recurrence due to skin sparing techniques [46]. However, research has proven a combination of skin sparing mastectomy and immediate reconstruction to have a low recurrence rate of 5.5% with invasive breast cancers and 0% for in-situ following a 6-year study [46].

Post-operatively the patient is transferred to a high dependency unit under the care of a specialised breast nurse who assesses the flap on an hourly basis for the first 24 hours as well as carrying out close monitoring of the patient’s vital observations, i.e. blood pressure, heart rate etc. The first 24–48 hours are crucial in early identification of any potential complications that could affect flap survival. Following this period, the patient is encouraged to sit up and gently mobilise with discharge planned 5–7 days post-operatively.

## Non-autologous breast reconstruction

Non-autologous reconstruction incorporates the use of implants which may be permanent depending on the shape and volume of the breast mound desired by the patient [17], [18], [44], [47], [48]. They can be silicone or saline filled, but the former is believed to be superior in creating a soft and aesthetically pleasing breast shape. Another alternative is the use of tissue expanders, which are placed under the pectoralis major muscle and inflated at weekly intervals to allow for expansion of the overlying tissues [31], [36]. This can then be replaced with a permanent implant once the desired volume is achieved [31], [36]. Although the use of breast implants can be advantageous in breast reconstruction as it provides an aesthetically pleasing result with a short recovery period, it is not recommended for patients undergoing post-mastectomy radiotherapy due to the potential risk of wound breakdown and extrusion [31], [32], [33], [47].

Predominantly, breast implants are associated with a risk of capsular contraction due to the formation of a thickened fibrous capsule which surrounds the implant (encapsulation) and causes a distortion of the breast shape as well as immense discomfort to the patient [47]. However, this is surgically correctable by successfully releasing the capsule and replacing the implant [48]. The lifespan of implants in the framework of breast reconstruction is limited, with silicone gel implants lasting approximately 16.4 years, and saline implants for 108 months [48].

The final stage of reconstruction involves recreating the nipple and areolar complex, which is considered an integral part of the procedure [49]. Local bilobed or tri-lobed skin flaps are raised and fashioned into a new nipple, and this procedure can be performed 4–6 months after the major reconstruction [49]. This can be followed at a later stage with tattooing to recreate the areolar complex [49]. Patients are also offered a modification of the contralateral breast if they are unhappy with the degree of asymmetry [50], [51]. This involves a mastopexy, reduction or augmentation with a breast implant for completion [50], [51].

## Breast cancer, breast reconstruction and COVID-19

Patients with cancer are at

1. an elevated risk for contracting COVID-19;
2. increased risk of a more severe infection; and
3. increased risk of developing complications and severe events related to COVID-19,

necessitating ICU admission with a higher mortality rate, due to their immunodepression, poor functional status, and frequent hospital visits and admissions [8], [9], [10], [52]. A study reported that oncologic patients who underwent surgery in the 30 days before contracting COVID-19 in China developed more frequently a severe form of disease compared to those who did not underwent surgery [53].

Breast reconstruction is associated with significantly improved quality of life and mental health after mastectomy [34], [35]. Breast reconstruction itself can fundamentally be classified into three classes [14] including

1. implant- and expander-based breast reconstruction,
2. flap-based breast reconstruction (vascularized autologous tissue), a combination of both (flap and implant), and
3. breast reconstruction using fat grafting (non-vascularized autologous lipoaspirate fat).

Mastectomy with immediate implant-based breast reconstruction has risen to be the most common method due to advances in meshes and implants [26], [45], [54] and has the advantages of improved body image, improved health-related quality of life and higher patient satisfaction compared to those who opt for delayed reconstruction [34], [35]. Pre-pectoral implant-based reconstruction in the delayed-immediate autologous reconstruction leads to significantly lower complication rates and shorter intervals between staged surgeries [36]. A systematic review of infection rates and acellular dermal matrix breast reconstruction found a higher infection rate associated with acellular dermal matrix reconstruction (11.59%) compared with the non- acellular dermal matrix patients (4.74%) [55]. Breast conservation surgery compared to the alternative of mastectomy has gained momentum following the results of large clinical trials which demonstrated equivalent long-term survival, despite a higher local recurrence [56], [57], [58]. In patient with mastectomy post breast conserving surgery and radiation therapy, delayed autologous microvascular breast reconstruction is a safer decision [34], [35], [36].

The onset of the COVID-19 pandemic has changed the face of the treatment of post-ablative breast reconstruction globally [52]. Reconstruction should still be discussed wherever possible, but thought should be given to minimising extent of surgery, stay in hospital and risk of complications during the pandemic. In line with South African government directives, our institution limits elective surgery to oncologic procedures, and the reconstructive time is still considered an integrated part of the treatment. Patients are evaluated case by case by a multidisciplinary team composed by breast surgeon, oncologist, pathologist, radiologist and plastic surgeon to minimize the exposure to COVID-19 without compromising oncological safety and offering the best possible aesthetic outcome. The function of the reconstructive surgeon is to attain a satisfactory aesthetic result. Reconstruction is discussed when probable, but thought is given to minimising operating times, in-hospital stay (day case and 24 hours stay procedures), risk of complications and number of out-patient visits (e.g., tissue expander inflation) during the pandemic to control the risk of SARS-CoV-2 exposure to the patient and health care staff [59], [60]. Only the breast with cancer is addressed to avoid prolonged surgery by avoiding concurrent contralateral balancing procedures. Neither prophylactic breast surgery for risk reducing mastectomies (e.g. for high genetic risk) nor its reconstruction should be performed until post COVID-19 pandemic. In addition, secondary, revision (e.g. fat grafting) and delayed post-ablative reconstruction are elective and therefore are be postponed until the local health care system has capacity for “safe“ elective surgery. Fat grafting is predominantly used to refine post-reconstructive asymmetries and should not be performed during the COVID-19 pandemic. Day case and 24 hours stay procedures can be performed but procedures requiring longer hospital admission should be avoided (e.g. free flap autologous breast reconstruction). Delayed reconstructive breast surgery post COVID-19 pandemic hypothetically offers the safest approach [61].

It is important for the patient to be fully informed about the risks and benefits of the procedure taking into consideration the risk of hospital acquired COVID-19 infection. At present, decision making may be within present ethical/practice standards and follow accepted guidelines and protocols [62], [63], [64], [65], [66], [67], [68], [69], [70], [71], [72]. However, should the burden on health services continue to escalate as has been seen in other countries decision making may be more extraordinary. Clear, open and transparent decision-making is also particularly important during the pandemic response [68], [69], [70], [71], [72]. In these conditions, it is critical that when the decision is made, both the decision process and decision made is well documented [68], [69], [70], [71], [72]. 

## Recommended procedures for breast reconstruction during COVID-19 pandemic

Immediate breast reconstruction using prepectoral implants or tissue expanders can continue after case-by-case multidisciplinary discussions of the patient’s individual risk factors (e.g. age, co-morbidities, radiation) and a change in future reconstructive options (i.e anticipated radiation therapy), or when conservation of the the skin envelope of the breast will facilitate a potential delayed autologous microvascular breast reconstruction. In addition, consideration must be given to the current availability of local health care system resources. This immediate breast reconstruction, by the prepectoral or tissue expander implant, at the time of the primary breast tumour resection leads to only slightly extended surgical times, but still preserves the breast skin which allows for a potential second step delayed autologous microvascular breast reconstruction.

Simple oncoplastic procedures including mammoplasty and integration of perforator flaps for volume replacement can be performed [73]. In fact ‘oncoplastic’ techniques to avoid the need for mastectomy should be encouraged to enhance less invasive surgery [73]. Palliative and salvage mastectomy procedures that expose the thoracic wall should be reconstructed immediately with local pivot flaps [73], [74].

Mastectomy scar placement should be discussed with the reconstructive surgeon in preparation for delayed oncoplastic procedures. Healthy mastectomy flaps will facilitate delayed and excision of excess skin should be limited except where viability is of concern. Placement of drains should preserve perforators and vessels to allow reconstructive flap options for possible delayed partial breast reconstruction. 

## Discussion

Breast reconstruction practice must be fluid and adapt to changing circumstances with close co-operation between breast and plastic surgeons working synergistically within a multidisciplinary team [70]. 

High level ACE2 gene expression has been demonstrated in skin, fat and breast tissue [8], [9], [10]. In addition, free flap failures have been associated with COVID-19 [11]. Post-ablative breast reconstruction is best kept straightforward and trouble-free during the COVID-19 pandemic. The pandemic is at different stages of the curve worldwide and resumption of post-ablative breast reconstruction practice should closely mirror local and national guidelines and exercise due caution to minimise risks of complications whilst addressing clinical need and patient expectations. Immediate breast reconstruction poses a unique surgical dilemma during COVID-19 as it straddles both urgent and elective forms of surgery. Expectations must be realistic with appropriate selection of patients and adherence to a fully informed consent process that reflects the additional risks associated with COVID-19. Individual risk assessment within an oncoplastic multidisciplinary team should help to risk stratify patients based on the latest data [75]. 

The existing data suggest that surgery may have a negative impact on the outcome of COVID-19 positive patients; however, there is currently insufficient evidence to deny patients the benefits of an immediate reconstruction. Reconstructive breast options will be restricted for the period of the COVID-19 pandemic, but there is opportunity to offer selected reconstructive options depending on local circumstances, operating capacity and the pandemic phase. We propose that breast reconstructive surgeons should consider only addressing the cancer side with immediate breast reconstruction using prepectoral “babysitter” implants or tissue expanders and greater use of therapeutic mammaplasty procedures that will only slightly extend surgical times. With the intention of counteracting some of the disadvantages of COVID-19 on breast cancer patients, breast conserving surgery should be considered when possible. Autologous microvascular breast reconstruction, complex oncoplastic procedures and all revisional breast reconstruction procedures should be recommenced safely once the peak of COVID-19 transmission has passed, as long as patients are suitably selected and appropriate modifications are considered. The recommendations considered in this article remain to be validated in future studies. 

## Competing interests

The authors declare that they have no competing interests.
